# Cross-regional cultural recognition of adolescent voice emotion

**DOI:** 10.3389/fpsyg.2024.1437701

**Published:** 2024-12-16

**Authors:** Shanshan Cheng, Yue Li, Yingying Wang, Yin Zhang

**Affiliations:** ^1^Department of Psychology, Shaoxing University, Shaoxing, Zhejiang, China; ^2^Center for Brain, Mind, and Education, Shaoxing University, Shaoxing, Zhejiang, China; ^3^Postdoctoral Research Station of Psychology, Henan University, Kaifeng, Henan, China

**Keywords:** emotion recognition, cross-cultural, in-group advantage, adolescents, cultural exposure

## Abstract

**Background:**

In previous studies, an in-group advantage in emotion recognition has been demonstrated to suggest that individuals are more proficient in identifying emotions within their own culture than in other cultures. However, the existing research focuses mainly on the cross-cultural variations in vocal emotion recognition, with limited attention paid to exploring intracultural differences. Furthermore, there is little research conducted on the ability of adolescents to recognize the emotions conveyed by vocal cues in various cultural settings. To fill these research gaps, three experiments were conducted in this study to explore the differences among different regions within a culture.

**Methods:**

The study involved three experiments. In Experiment 1, a within-subjects design of 2 (language: Mandarin vs. English) × 4 (emotion: anger vs. fear vs. happiness vs. sadness) was used to establish whether adolescents exhibit a similar in-group advantage in vocal emotion recognition with adults. As an expansion of Experiment 1, Experiment 2 incorporated the Shaoxing dialect to assess the ability of adolescents to identify the emotions in voices across different cultural regions of a nation. In Experiment 3, the regional variation was extended by substituting the Shaoxing dialect with Tibetan to explore the disparities in vocal emotion recognition among adolescents.

**Results:**

As indicated by the results of Experiment 1, Mandarin**-**speaking adolescents performed well in recognizing emotions in Mandarin compared to English. In Experiment 2, the results of Experiment 1 were replicated to reveal that Shaoxing**-**speaking adolescents performed better in emotion recognition of Mandarin in comparison to the Shaoxing dialect and English. As indicated by Experiment 3, both Mandarin**-**speaking adolescents and Tibetan**-**speaking adolescents possessed a higher capacity of vocal emotion recognition within their own language groups.

**Conclusion:**

Chinese adolescents demonstrated a stronger ability to recognize vocal emotions within their own cultural group compared to other regional cultures, an advantage that became more pronounced as the cultural differences between groups increased. These findings underscore the significance of cultural factors in adolescent emotional recognition research, indicating the directions of cross-cultural interventions.

## 1 Introduction

Defined as the skill of accurately identifying and distinguishing emotional states through observation of the valid cues in others’ behavior, emotion recognition plays a crucial role in everyday social interactions (Cotter et al., 2018; Spunt and Adolphs, 2019). This skill is essential for dealing with the complexities within human communication, as it enables individuals to respond appropriately to the emotional expressions of others, thus facilitating empathy and cooperation. As a specific form of emotion recognition, voice emotion recognition focuses on examining the unique acoustic features in a speaker’s voice to recognize their emotional state. These features include pitch, volume, tempo, voice quality and intonation patterns, all of which are useful to convey critical information about the speaker’s emotional tone. As online vocal communication becomes more frequent, voice emotion recognition plays an increasingly important role in human social interaction.

Some studies have found that the constrained metrical space in language can hinder tone languages, such as Chinese, in effectively conveying emotions compared to non-tone languages, such as English (Ross et al., 1986; Cruttenden, 1997; Ip and Cutler, 2020; Schwarz et al., 2023). With tones used for lexical distinctions, tone languages may involve the limited use of paralanguage for emotional expression, which may compromise the accuracy in recognizing vocal emotions. Conversely, non-tone languages are more flexible in manipulating intonational contours, which allows for more nuanced emotional expression (Zhu, 2013). However, the in-group advantage holds that individuals are more adept at discerning vocal emotions from members of their own cultural group. This concept underscores the significant role of culture in shaping emotional expression, with the perception of emotions potentially influenced by geographical and dialectal variations (Paulmann and Uskul, 2014). This is also related to cultural exposure, as the recognition and interpretation of emotions within and across cultural boundaries can be modulated by the degree and nature of exposure to different cultures (Elfenbein and Ambady, 2003a; Elfenbein and Ambady, 2003b). There is a large amount of evidence demonstrating that cultural exposure exerts influence on emotion recognition across different cultures. Native speakers and second language speakers differ in the expression of certain emotions (Liu et al., 2015).

Despite the universal systems of emotional expression, some degree of cross-cultural recognition is enabled, with the unique emotional expression patterns of each culture sculpted by a confluence of cultural traditions, social norms, and educational practices (Laukka et al., 2014). The empirical research on cross-cultural vocal emotion recognition is consistent in advocating for the in-group advantage (Albas et al., 1976; Scherer et al., 2001; Sauter, 2013; Laukka et al., 2014; Laukka and Elfenbein, 2021). Nevertheless, there are few comparative studies conducted between tone languages and non-tone languages. Therefore, it is crucial to conduct research in this regard, especially considering the further exploration of emotional recognition among various dialects within spoken languages.

The current research focuses mainly on the emotion recognition across regional cultures within the same nation. As the second-largest language family with the highest number of speakers, Chinese exhibits unique characteristics as a tone language (Wang et al., 2014).

Mandarin, which represents the dominant language in China, Wu language (with Shaoxing dialect being one of its variants) as a significant regional variety, and Tibetan, an important ethnic language, all belong to the Sino-Tibetan language family. Similar to Mandarin, the Shaoxing dialect exhibits four tonal patterns. However, in comparison, the Shaoxing dialect potentially shows a greater diversity of falling and rising tonal variations, and the pitch fluctuations within the same tonal classification are more intricate (Li and Yang, 2024). Tibetan, likewise, involves four tones. Although the pronunciations of the first, second, and fourth tones in Tibetan bear resemblance to those in Mandarin, the third tone (the upper tone) in Tibetan differs from its counterpart in Mandarin. Tibetan is an inflected language, featuring a rich array of vowels and consonants, with special laryngeal sounds included (Cui and Zhang, 2009). Under the context of Chinese culture, a substantial research gap to fill is to dissect the cross-regional cultural variances in the perception of emotions.

There remains uncertainty in the extent to which voice emotion recognition is consistent between adolescents and adults. Although it has been established in prior studies that Chinese adults possess an in-group advantage in cross-cultural vocal emotion recognition, the research on this phenomenon among adolescents is still limited. According to the study conducted by Vidas et al. (2018), children aged 8 or over demonstrate comparable abilities to adults in identifying musical emotions. It has been shown in other studies that the capability to recognize emotions may vary depending on the specific emotion and age group. For instance, it is suggested in the research of Chronaki et al. (2015) that the recognition of sadness and happiness in non-verbal vocalizations matures earlier than the recognition of anger and fear. However, the overall performance in emotion recognition typically reaches adult levels by the age of 15 (Grosbras et al., 2018). Furthermore, Amorim et al. (2021) investigated the changes in vocal emotions recognition across different age groups, from children to the elderly. It was revealed that the accuracy in vocal emotion recognition shows improvement from childhood to early adulthood but decline in older age groups. Focusing on the adolescents aged 12–15, this study determined if they also exhibit a similar advantage in recognizing emotions within their age group, as seen in adults under cross-cultural contexts.

The objective of this study is to explore the existence of an in-group advantage in the recognition of vocal emotions among adolescents from diverse Chinese cultural backgrounds. To achieve this objective, an investigation will be conducted using Mandarin, Shaoxing dialect, and Tibetan dialect as experimental stimuli for assessment of the cultural distance in emotion recognition accuracy.

## 2 Experiment 1: cross-cultural voice emotions recognition in adolescents

### 2.1 Methods

#### 2.1.1 Participants

The experiment was conducted by randomly recruiting 87 middle school students (43 boys, 44 girls) aged between 13 and 15 years old, with an average age of 13.98 years. The primary language spoken by the participants was Mandarin. Supplementary Table 2 can be referred to for further details regarding participant information and language proficiency. All the participants had normal vision or corrected vision and normal hearing. Those with mental illnesses, severe psychological disorders, autism, or other emotional recognition disorders were excluded. Participants were also given a gift as a token of appreciation following the experiment. Prior to the study, informed consent was obtained from both the participants and their parents. Conducted in strict accordance with the applicable ethical standards, this study was approved by the Shaoxing University Ethics Committee (No. 2023–060-01). G*Power 3.1 software was applied to calculate a two-factor repeated measures ANOVA with a statistical power of 80%, a significance level of 0.05, an effect size of 0.25 (medium scale according to Cohen, 2013), and a minimum sample size of 48.

#### 2.1.2 Materials

The audio materials used in Experiment 1 were carefully selected according to the previous research conducted by Paulmann and Uskul (2014) and Liu and Pell (2012). To ensure the accuracy and consistency of emotions being expressed, these studies had been thoroughly recorded and rated. Four fundamental emotions (anger, fear, happiness, and sadness) were involved in this study. Moreover, the audio materials consist of the pseudo sentences that follow grammatical rules but lack any inherent meaning or emotional connotations. More details are provided by Supplementary Table 1. The sentences in English are cited completely from the research conducted by Paulmann and Uskul (2014), while the Mandarin sentences are translations of the English sentences. The Mandarin sentences were acted out by two individuals experienced in dubbing, with different emotions performed. The duration of both the English and Mandarin recordings ranged from 2000 ms to 3,000 ms. Post-processing was carried out on the Mandarin recordings, with volume adjustment made to ensure a standardized listening level for the participants during emotion recognition tasks. Each sound sample was saved as a separate .wav file with a frequency of 44,100 Hz, while monophonic 16-bit sampling was performed to maintain the consistency in sound quality.

In total, 140 English sentences and 160 Chinese sentences were selected and produced, followed by the assessment of them. To ensure emotional relevance, 40 native Chinese-speaking secondary school students participated in the assessment of audio materials. To avoid biased scoring, the order of the audio materials was randomized. The utterances with a recognition accuracy above 70% were used. A total of 80 recordings (40 in English and 40 in Mandarin) were finalized as the formal experimental materials. The specific recording materials for the formal experiment can be accessed via the following link: https://github.com/chengshanshan123/Experimental-data.

#### 2.1.3 Design and procedure

Experiment 1 involved a 2 (language: Mandarin, English) × 4 (emotion: anger, fear, happiness, sadness) within-subjects design, with the dependent variables as the emotion recognition accuracy and response time of the participants.

The experiment was conducted using a 16-inch laptop to present the stimuli through headphones. E-prime 2.0 software was applied for stimulus presentation and response time recording. The experimental environment was kept quiet. The participants performed an emotion recognition task with their eyes open throughout. They were instructed that it was not necessary to understand the audio content and their judgments should be based solely on the emotions conveyed in the sounds. Before the formal experiment began, the participants were required to familiarize themselves with the procedure and response keystrokes through 8 practice trials. The practice phase had the same procedure and stimuli presentation as the formal experiment, with feedback received by the participants.

**Figure 1:**
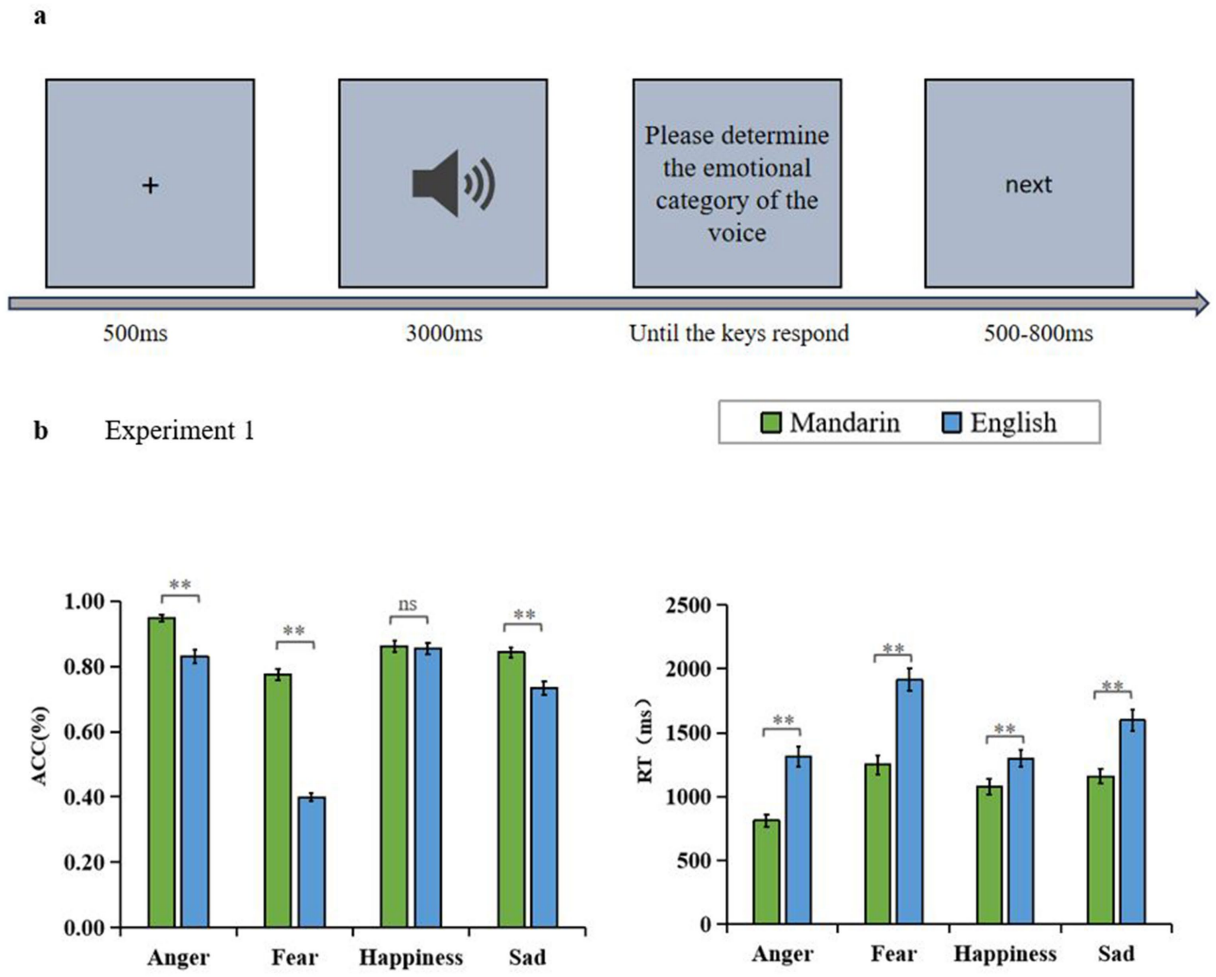
**(A)** The experimental procedure in Experiment 1, 2, 3. **(B)** Results of Experiment 1, accuracy rate and response time for cross-cultural voice emotion recognition in Mandarin-speaking adolescents.

During the experiment, the participants were assigned specific keys (A, S, D, F) to correspond with different emotions (anger, fear, happiness, sadness). To control bias, these key-emotion mappings varied among the participants. The experiment consisted of two blocks, each with 40 trials. Thus, a total of 80 trials were involved. There was a 2-min break between the two blocks. Auditory stimuli were presented randomly, each of which lasted 3,000 ms and was played once. The participants were asked to select the corresponding emotion category by pressing a key after each sound. Once a choice was made, the program proceeded, with accuracy and response time recorded. The participants were instructed to prioritize accuracy in their responses. Figure 1A illustrates the process of stimulus presentation.

### 2.2 Results

A two-factor repeated measures ANOVA was conducted on the data (response time and accuracy) using SPSS 27.0 software to investigate the effects of language (Mandarin and English) and vocal emotion (anger, fear, happiness, and sadness). Post-hoc comparisons were performed in case of a significant main effect, followed by the analyses of significant interactions and subsequent simple effects. The Greenhouse–Geisser method was used to correct *p*-values, and partial eta-squared ( $\eta_{p}^{2}$ ) was calculated to measure the effect size.

#### 2.2.1 Accuracy rate (ACC)

The ACC results revealed a significant main effect of language [*F* (1, 86) = 230.715, *p* < 0.01, $\eta_{p}^{2}$ = 0.728]. Post-hoc analysis showed that participants’ emotion recognition scores were significantly higher for Mandarin materials (M = 0.857, *SD* = 0.010) compared to English materials (M = 0.704, *SD* = 0.010), *p* < 0.01. The main effect of voice emotion categories was also significant [*F* (3, 258) = 132.730, *p* < 0.01, $\eta_{p}^{2}$ = 0.607]. Post-hoc analysis revealed that the highest recognition scores were for anger (M = 0.889, *SD* = 0.014) and happiness (M = 0.858, *SD* = 0.014), with no significant difference between them, *p* > 0.05. Sadness scored significantly lower than happiness and anger (M = 0.787, *SD* = 0.014), *p* < 0.01, Fear scored significantly lower than sadness (M = 0.587, *SD* = 0.012), *p* < 0.01. There was a significant interaction between language and emotion categories [*F* (3, 258) = 53.026, *p* < 0.01, $\eta_{p}^{2}$ = 0.381]. Simple effects analyses indicated that participants recognized Mandarin more accurately than English for all voice emotion categories, with significant differences for anger, fear, and sadness [*F* (1, 86) = 33.418, *p* < 0.01, $\eta_{p}^{2}$ = 0.280; *F* (1, 86) = 326.304, *p* < 0.01, $\eta_{p}^{2}$ = 0.791; *F* (1, 86) = 21.183, *p* < 0.01, $\eta_{p}^{2}$ = 0.198]. There was no significant difference in happiness, *p* = 0.683 (Figure 1B).

#### 2.2.2 Response time (RT)

The RT results revealed a significant main effect of language [*F* (1, 86) = 128.171, *p* < 0.01, $\eta_{p}^{2}$ = 0.598]. Post-hoc analysis indicated that the response times for Mandarin (M = 1074.054, *SD* = 47.761) were significantly faster than for English (M = 1530.409, *SD* = 60.827), *p* < 0.01. There was also a significant main effect of emotion categories [*F* (3, 258) = 35.376, *p* < 0.01, $\eta_{p}^{2}$ = 0.291]. Post-hoc analysis showed that anger was recognized as the fastest among different emotion categories (M = 1061.178, *SD* = 53.253), followed by happiness (M = 1188.879, *SD* = 63.799), and significantly slower for fear (M = 1582.015, *SD* = 74.407) and sadness (M = 1376.854, *SD* = 58.422), *p* < 0.01. Furthermore, there was a significant interaction between language and emotion categories [*F* (3, 258) = 7.738, *p* < 0.01, $\eta_{p}^{2}$ = 0.083]. Simple effects analyses showed that Mandarin was significantly faster than English when responding to each of the four voice emotion categories: anger *F* (1, 86) = 61.141, *p* < 0.01, $\eta_{p}^{2}$ = 0.416, fear *F* (1, 86) = 94.982, *p* < 0.01, $\eta_{p}^{2}$ = 0.525, happiness *F* (1, 86) = 9.710, *p* < 0.01, $\eta_{p}^{2}$ = 0.101, and sadness *F* (1, 86) = 32.059, *p* < 0.01, $\eta_{p}^{2}$ = 0.272 (Figure 1B).

### 2.3 Discussion

As indicated by the results of Experiment 1, compared with the recognition of emotions expressed in English, Mandarin-speaking adolescents possessed a significant advantage in identifying the vocal emotional expressions in Mandarin, including various emotions such as anger, fear, happiness, and sadness. This advantage implies the potential challenges in processing cross-cultural emotional expressions. The differences in culture, pronunciation, and intonation may explain why it takes more cognitive effort and time to understand and distinguish emotions in English.

As revealed by Paulmann and Uskul (2014), Chinese adults were more adept at identifying emotions when expressed in Chinese compared to English. Yan et al. (2022) also found out that Mandarin-speaking adults recognized the emotions in Mandarin more accurately than in English. The present study on adolescents aligns with these previous findings on adults.

Based on the findings of Experiment 1, Experiment 2 was performed to establish whether there is an in-group advantage in recognizing emotions through vocal emotions among Mandarin adolescents and Shaoxing adolescents. Both groups share the same language family and national culture. Specifically, Experiments 2a and 2b were aimed at assessing the ability of these adolescents to recognize the emotions in Mandarin and other Chinese dialects (such as the Shaoxing dialect), respectively. This is conducive to determining whether the in-group advantage remains consistent across different regions of the same overarching culture.

## 3 Experiment 2: intra-cultural voice emotions recognition in adolescents

### 3.1 Methods

#### 3.1.1 Materials

The English and Mandarin sentences used in Experiment 1 were repeated in this experiment. The Shaoxing dialect was recorded using the same set of sentences as those for the Mandarin recordings, but spoken in Shaoxing dialect. In total, 120 recordings (40 in English, 40 in Mandarin, and 40 in Shaoxing dialect) were finalized as the formal experimental materials. More details are provided by Supplementary Table 1 and the link: https://github.com/chengshanshan123/Experimental-data.

#### 3.1.2 Design and procedure

Experiment 2 was carried out using a 3 (language: Mandarin, Shaoxing, English) × 4 (emotion: anger, fear, happiness, sadness) within-subjects design, with accuracy and response time measured as dependent variables. The experimental procedure is the same as in Experiment 1.

### 3.2 Experiment 2a: emotional recognition of intra-cultural voices by Mandarin adolescents

#### 3.2.1 Participants

The recruitment process for participants in Experiment 2a is identical to that of Experiment 1. All participants are native Mandarin speakers, coming from areas where Mandarin is the primary language spoken. A total of 44 middle school students (consisting of 19 boys and 25 girls, aged 13–15 years with an average age of 14 years) were recruited. Prior to the study, informed consent was obtained from both the participants and their parents. The experimental design was approved by the Shaoxing University Ethics Committee (No. 2023–060-01). More details of participant information and language proficiency are provided by Supplementary Table 2.

#### 3.2.2 Statistical analysis

Same as Experiment 1.

#### 3.2.3 Results

##### 3.2.3.1 Accuracy rate (ACC)

**Figure 2:**
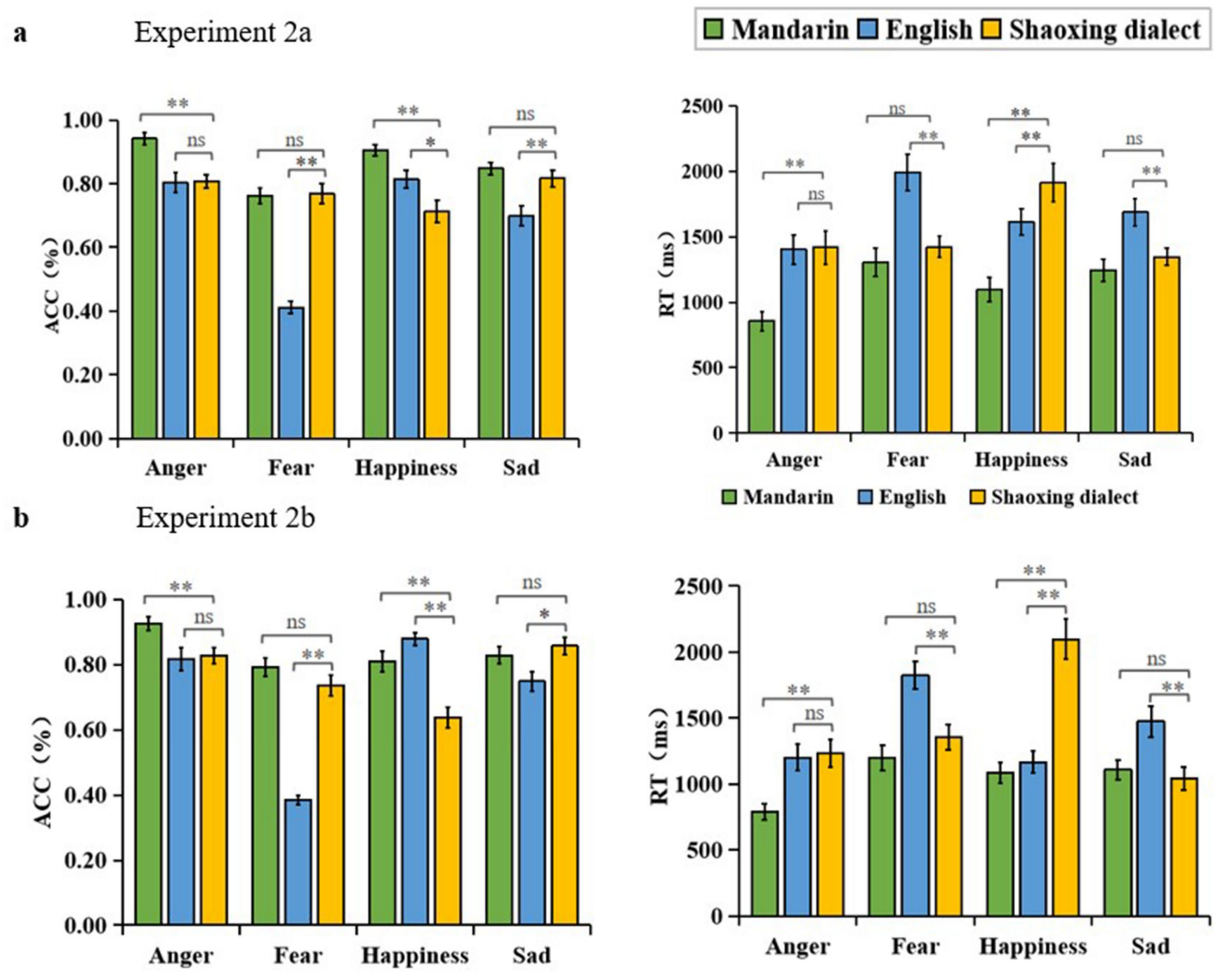
**(A)** Results from Experiment 2a, accuracy rates and response times of Mandarin-speaking adolescents’ cross regional cultural voice emotion recognition. **(B)** Results of Experiment 2b, accuracy rate and reaction time of cross-cultural voice emotion recognition across regions for Shaoxing-speaking adolescents.

The ACC results revealed a significant main effect of language [*F* (2, 86) = 89.742, *p* < 0.01, $\eta_{p}^{2}$ = 0.676]. Post-hoc analysis showed that participants’ emotion recognition scores for Mandarin (M = 0.864, *SD* = 0.013) were significantly higher than those for Shaoxing dialect (M = 0.776, *SD* = 0.019), and Shaoxing dialect scores were significantly higher than those for English (M = 0.682, *SD* = 0.017), *p* < 0.01. There was a significant main effect of emotion categories [*F* (3, 129) = 34.533, *p* < 0.01, $\eta_{p}^{2}$ = 0.445]. Post-hoc analysis showed that participants had the highest scores for recognizing the different emotion categories for anger (M = 0.850, *SD* = 0.019) and happiness (M = 0.811, *SD* = 0.022), and that there was no significant difference between the anger and happiness, *p* > 0.05, and scores significantly lower than anger were sadness (M = 0.788, *SD* = 0.018), *p* < 0.01 and fear (M = 0.647, *SD* = 0.018), *p* < 0.01. The interaction between language and emotion were significant [*F* (6, 258) = 20.797, *p* < 0.01, $\eta_{p}^{2}$ = 0.326]. Simple effects analyses indicated that participants recognized anger, happiness, and sadness better in Mandarin than in Shaoxing, with a significant difference in anger and happiness, anger *F* (2, 42) = 23.500, *p* < 0.01, $\eta_{p}^{2}$ *^2^* = 0.528; happiness *F* (2, 42) = 26.599, *p* < 0.01, $\eta_{p}^{2}$ = 0.559. There was no significant difference in fear (*p* = 1.000) and sadness (*p* = 0.753) (Figure 2A).

##### 3.2.3.2 Response time (RT)

The RT results revealed a significant main effect of sound material language on response time [*F* (2, 86) = 62.459, *p* < 0.01, $\eta_{p}^{2}$ = 0.592]. Post-hoc analysis showed that the response times for Mandarin material (M = 1124.673, *SD* = 73.790) were significantly faster than for Shaoxing dialect (M = 1524.657, *SD* = 80.229), *p* < 0.01. Shaoxing dialect was significantly faster than English material (M = 1672.741, *SD* = 87.073), *p* < 0.05. There was a significant main effect of response time for emotion categories [*F* (3, 129) = 10.754, *p* < 0.01, $\eta_{p}^{2}$ = 0.200]. Post-hoc analysis showed that participants had the fastest response time for the different emotion categories recognized, with anger (M = 1226.130, *SD* = 91.867), and the next highest response time above anger was sadness (M = 1424.939, *SD* = 63.849), *p* < 0.05, and scoring significantly slower than anger were happiness (M = 1539.316, *SD* = 84.947) and fear (M = 1572.377, *SD* = 98.020), *p* < 0.01. There was a significant interaction between language and emotion [*F* (6, 258) = 8.062, *p* < 0.01, $\eta_{p}^{2}$ = 0.158]. Simple effects analyses indicated that Mandarin-speaking adolescents were faster than Shaoxing in recognition response time for all four emotions, with significant differences in anger and happiness, anger *F* (2, 42) = 26.987, *p* < 0.01, $\eta_{p}^{2}$ = 0.562, and happiness *F* (2, 42) = 21.160, *p* < 0.01. There was a significant difference in the interaction between fear (*p* = 0.408) and sadness (*p* = 0.665) the differences were not significant (Figure 2A).

### 3.3 Experiment 2b: emotional recognition of intra-cultural voices by Shaoxing adolescents

#### 3.3.1 Participants

The recruitment process for participants in Experiment 2b was identical to that of Experiment 2a. The only difference is that all the participants are the native speakers of the Shaoxing dialect who come from the area where the Shaoxing dialect is commonly spoken. A total of 51 students (28 boys and 23 girls) were recruited, with an age range of 12–15 years and a mean age of 12.61 years. Informed consent was obtained from both the participants and their parents prior to the study. The experimental design was approved by the Shaoxing University Ethics Committee (no. 2023-060-01). Supplementary Table 2 provides more details of participant information and language proficiency.

#### 3.3.2 Statistical analysis

Same as experiment 2a.

#### 3.3.3 Results

##### 3.3.3.1 Accuracy rate (ACC)

The ACC results revealed a significant main effect of language [*F* (2, 90) = 33.988, *p* < 0.01, $\eta_{p}^{2}$ = 0.430]. Post-hoc analysis showed that emotion recognition scores for Mandarin material (M = 0.839, *SD* = 0.016) were significantly higher than those for Shaoxing dialect (M = 0.764, *SD* = 0.021), and Shaoxing dialect scores were significantly higher than those for English (M = 0.707, *SD* = 0.015), *p* < 0.01. The main effect of emotion categories was significant [*F* (3, 135) = 38.193, *p* < 0.01, $\eta_{p}^{2}$ = 0.459]. Post-hoc analysis showed that participants had the highest scores for anger (M = 0.857, *SD* = 0.023) and sadness (M = 0.811, *SD* = 0.019) in recognizing different emotion categories, which were not significantly different from each other, with *p* > 0.05. Scores significantly lower than anger and sadness was fear (M = 0.638, *SD* = 0.013), *p* < 0.01. Happiness (M = 0.775, *SD* = 0.018) was significantly lower than anger, *p* < 0.01, and significantly higher than fear, *p* < 0.01. The interaction between language and emotion categories was significant [*F* (6, 270) = 33.470, *p* < 0.01, $\eta_{p}^{2}$ = 0.427]. Simple effects analyses showed that Shaoxing-speaking adolescents recognized Mandarin more than Shaoxing dialect on anger, fear, and happiness, with significant differences on anger and happiness, anger *F* (2, 44) = 11.200, *p* < 0.01, $\eta_{p}^{2}$ = 0.337; happiness *F* (2, 44) = 26.116, *p* < 0.01, $\eta_{p}^{2}$ = 0.543. The difference was not significant on fear (*p* = 0.147) and sadness (*p* = 1.000) (Figure 2B).

##### 3.3.3.2 Response time (RT)

The RT results revealed a significant main effect of sound material language on response time [*F* (2, 180) = 52.576, *p* < 0.01, $\eta_{p}^{2}$ = 0.369]. Post-hoc analysis showed that the response times for Mandarin material (M = 1140.554, *SD* = 48.926) were significantly lower than for Shaoxing dialect (M = 1610.973, *SD* = 79.440), *p* < 0.01. There was no difference between Shaoxing and English materials (M = 1608.134, *SD* = 71.044), *p* > 0.05. The main effect of emotion categories on response time was significant. Emotions had a significant main effect [*F* (3, 270) = 25.957, *p* < 0.01, $\eta_{p}^{2}$ = 0.224]. Post-hoc analysis showed that participants had the lowest response times for anger (M = 1188.429, *SD* = 61.143), and significantly slower than anger were fear (M = 1679.586, *SD* = 77.510) and happiness (M = 1581.465, *SD* = 75.186), followed by sadness (M = 1363.403, *SD* = 69.549), *p* < 0.05. The interaction between voice material language and voice emotion categories was significant [*F* (6, 540) = 30.259, *p* < 0.01, $\eta_{p}^{2}$ = 0.252]. Simple effects analyses showed that on the anger, fear, and happiness, Shaoxing-speaking middle school students responded to Mandarin recognition tasks more quickly than they did to tasks in the Shaoxing dialect, with significant differences on anger and happiness, anger *F* (2, 44) = 16.840, *p* < 0.01, $\eta_{p}^{2}$ = 0.434; and happiness *F* (2, 44) = 21.407, *p* < 0.01, $\eta_{p}^{2}$ = 0.493. The difference for fear (*p* = 0.270) and sadness (*p* = 1.000) the difference was not significant (Figure 2B).

##### 3.3.3.3 Mandarin and Shaoxing participants comparison in Experiment 2

As for the results of ACC, a repeated measures ANOVA was conducted on Experiments 2a and 2b with participant group, language and emotion categories as independent variables and accuracy as the dependent variable. The analysis revealed, the main effect of group is insignificant [*F* (1, 88) = 0.036, *p* = 0.850, and $\eta_{p}^{2}$ = 0.990].

As for the results of RT, a repeated measures ANOVA was conducted on Experiments 2a and 2b with participant group, language and emotion categories as independent variables and response time as the dependent variable. The analysis indicated, the main effect of group is insignificant [*F* (1, 88) = 1.590, *p* = 0.215, and $\eta_{p}^{2}$ = 0.017].

### 3.4 Discussion

In Experiment 2, the emotional voices in the Shaoxing dialect were used to explore how Mandarin-speaking adolescents and Shaoxing-speaking adolescents perceive vocal emotions in various regions within a single country. This research aims to determine if there is an in-group advantage within a large and unified culture.

As observed in Experiment 2a, Mandarin-speaking adolescents demonstrated a significantly better ability to identify vocal emotions in Mandarin than in Shaoxing and English. This finding supports the hypothesis proposed in the experiment, confirming the prediction of the in-group advantage that individuals are adept at recognizing emotional expressions within their own cultural or linguistic group. Additionally, it took more time for the participants to identify the emotional sounds in English than in Shaoxing dialect. This finding aligns with the research conducted by Laukka and Elfenbein (2021), which indicates that the more pronounced in-group advantage is often attributed to a wider cultural gap between languages. This is because individuals are more familiar with the cultural connotations and emotional expressions within their own cultural-linguistic group. Additionally, a greater difference from other languages renders this familiarity advantage more prominent in the process of emotional recognition.

The results of Experiment 2b are inconsistent with our prediction. It was found out that Shaoxing-speaking adolescents exhibited a degree of advantage in recognizing Mandarin emotions compared to Shaoxing emotions. This might result from the fact that Mandarin is commonly used in educational and daily situations. There are many studies demonstrating the positive impact of language exposure on emotion recognition (Schwering et al., 2021; Barrett et al., 2007). Schwering et al. (2021) emphasized the exposure to new reading materials and the capacity to recognize emotions, revealing how linguistic experiences play a crucial role in the development of emotional comprehension. Although Mandarin and Shaoxing dialect are both part of the same culture, there are still variations in their linguistic structures and usage patterns. People who speak the Shaoxing dialect may use Mandarin more frequently for communication. As indicated by this difference, the amount and type of language exposure can directly affect one’s ability to recognize emotions, rather than just their linguistic or cultural background. In conclusion, the regular exposure to Mandarin has a potential in allowing Shaoxing adolescents to better understand and interpret the emotions expressed in Mandarin, even though Shaoxing dialect is their native dialect.

In Experiment 2, the adolescents from Shaoxing failed to show an in-group advantage, which may be due to Mandarin is already widely spoken throughout the Shaoxing region. Therefore, our aim is to find a region that is less influenced by Mandarin. Consequently, in Experiment 3, Tibetan was used, as Tibetan and Mandarin belong to the same language family. Therefore, it can be investigated whether the in-group advantage effect is observable within the same country. Also, it can be ascertained whether the in-group advantage exists across different regions of the same overarching culture.

## 4 Experiment 3: voice emotions recognition for adolescents to expand the cultural distance

### 4.1 Methods

#### 4.1.1 Materials

The English and Mandarin sentences from Experiment 1 were also used in this experiment. Tibetan dialect recordings used the same set of sentences as the Mandarin recordings. The Tibetan sentences were spoken by two native speakers of the Tibetan dialect. In total, 120 recordings (40 in English, 40 in Mandarin, and 40 in Tibetan dialect) were selected as the formal experimental materials. More details are provided by Supplementary Table 1 and the link: https://github.com/chengshanshan123/Experimental-data.

#### 4.1.2 Design and procedure

Experiment 3 was performing using a 3 (language: Mandarin, Tibetan, English) × 4 (emotion: anger, fear, happiness, sadness) within-subjects design, with accuracy and response time as dependent variables. The same experimental procedure as in Experiment 2 was adopted. In Experiment 3, the regional variation was extended by substituting the Shaoxing dialect with Tibetan to explore the differences in vocal emotion recognition among adolescents.

### 4.2 Experiment 3a: voice emotion recognition of Mandarin adolescents: an expansion of cultural distance

#### 4.2.1 Participants

The recruitment process for participants in Experiment 3a was identical to that of Experiment 2. All the participants are native Mandarin speakers with no proficiency in Tibetan. A total of 51 middle school adolescents (consisting of 28 boys and 23 girls, aged 12–15 years with an average age of 12.61 years) were recruited for this experiment. Prior to the study, informed consent was obtained from both participants and their parents. The experimental design was approved by the Shaoxing University Ethics Committee (no. 2023-060-01). The Supplementary Tables 1, 2 provide additional details on participant information and language proficiency.

#### 4.2.2 Statistical analysis

Same as Experiment 2.

#### 4.2.3 Results

##### 4.2.3.1 Accuracy rate (ACC)

**Figure 3:**
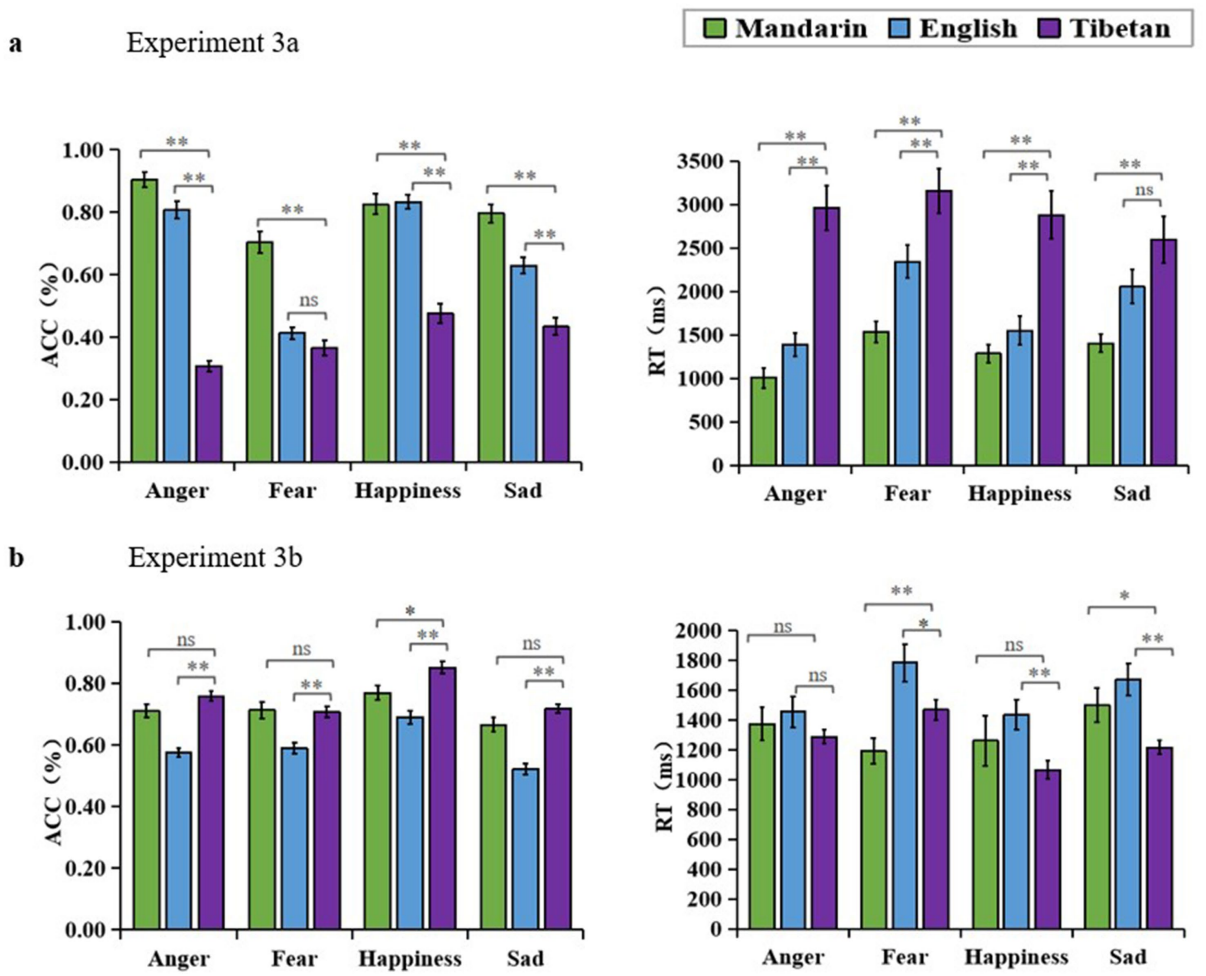
**(A)** Experiment 3a results, accuracy rate and response time of extended regional distance sound emotion recognition in Mandarin-speaking adolescents. **(B)** Experiment 3b results, accuracy rate and response time of Tibetan-speaking adolescents expanded regional distance voice emotion recognition.

The ACC results revealed a significant main effect of language [*F* (2, 100) = 354.067, *p* < 0.01, $\eta_{p}^{2}$ = 0.876]. Post-hoc analysis showed that emotion recognition scores for Mandarin material (M = 0.807, *SD* = 0.016) were significantly higher than those for Tibetan (M = 0.396, *SD* = 0.014) and English (M = 0.671, *SD* = 0.014), *p* < 0.01. The main effect of emotion categories was significant [*F* (3, 150) = 40.201, *p* < 0.01, $\eta_{p}^{2}$ = 0.446]. Post-hoc analysis showed that participants scored the highest on the recognition of different emotion categories for happiness (M = 0.712, *SD* = 0.021), and lower than happiness was anger (M = 0.673, *SD* = 0.014) no significant difference. Scoring significantly lower than happiness were sadness (M = 0.642, *SD* = 0.013), *p* < 0.01, and fear (M = 0.438, *SD* = 0.011), *p* < 0.01. The interaction between language and emotion categories was significant [*F* (6, 300) = 15.949, *p* < 0.01, $\eta_{p}^{2}$ = 0.242]. Simple effects analyses showed that the participants recognized Mandarin better than Tibetan on all four voice emotion categories, anger *F* (2, 49) = 269.675, *p* < 0.01, $\eta_{p}^{2}$ = 0.917; fear *F* (2, 49) = 31.880, *p* < 0.01, $\eta_{p}^{2}$ = 0.565; and happiness *F* (2, 49) = 58.898, *p* < 0.01, $\eta_{p}^{2}$ = 0.706; sadness *F* (2, 49) = 37.965, *p* < 0.01, $\eta_{p}^{2}$ = 0.608 (Figure 3A).

##### 4.2.3.2 Response time (RT)

The RT results revealed a significant main effect of language [*F* (2, 100) = 59.324, *p* < 0.01, $\eta_{p}^{2}$ = 0.543]. Post-hoc analysis showed that emotion recognition for Mandarin (M = 1306.879, *SD* = 79.333) was significantly faster than English (M = 1833.023, *SD* = 136.131), and English was significantly faster than Tibetan (M = 2897.835, *SD* = 218.076), *p* < 0.01. There was a significant main effect of emotion categories [*F* (3, 150) = 8.839, *p* < 0.01, $\eta_{p}^{2}$ = 0.150]. Post-hoc analysis revealed that, among the different recognized emotion categories, participants responded fastest to anger (M = 1785.708, SD = 132.560). Fear (M = 2342.976, SD = 155.724) elicited a significantly slower response than anger (*p* < 0.01). Sadness (M = 2017.643, SD = 152.637) and happiness (M = 1807.351, SD = 93.830) did not show significant differences from anger (*p* > 0.05). Happiness was significantly faster than fear (M = 2342.976, SD = 155.724) (*p* < 0.01). Sadness was significantly faster than fear (*p* < 0.05) and did not have significant differences compared to other emotions. There was a significant interaction between language and emotion categories [*F* (6, 300) = 10.331, *p* < 0.01, $\eta_{p}^{2}$ = 0.072]. Simple effects analyses showed that participants were significantly faster for Mandarin than for Tibetan for all four voice emotions: anger *F* (2, 49) = 47.494, *p* < 0.01, $\eta_{p}^{2}$ = 0.660; fear *F* (2, 49) = 26.585, *p* < 0.01, $\eta_{p}^{2}$ = 0.520; happiness *F* (2, 49) = 22.554, *p* < 0.01, $\eta_{p}^{2}$ = 0.479; sadness *F* (2, 49) = 17.231, *p* < 0.01, $\eta_{p}^{2}$ = 0.413 (Figure 3A).

### 4.3 Experiment 3b: voice emotions recognition for Tibetan-speaking adolescents: an expansion of cultural distance

#### 4.3.1 Participants

The recruitment process for participants in Experiment 3b was identical to that of Experiment 3a. The only difference is that all of the participants are native speakers of the Tibetan and come from Tibet. Although their Mandarin and English vocabulary is limited, they cannot communicate effectively. A total of 68 students (37 boys and 31 girls) were recruited, with an age range of 14.1 years and a mean age of 12.61 years. Before the study, informed consent was obtained from both participants and their parents. The experimental design was approved by the Shaoxing University Ethics Committee (No. 2023–060-01).

#### 4.3.2 Statistical analysis

Same as Experiment 3a.

#### 4.3.3 Results

##### 4.3.3.1 Accuracy rate (ACC)

The ACC results revealed a significant main effect of language [*F* (2, 134) = 36.813, *p* < 0.01, $\eta_{p}^{2}$ = 0.355]. Post-hoc analysis showed that emotion recognition scores for Tibetan (M = 0.759, *SD* = 0.012) were significantly higher than those for English (M = 0.594, *SD* = 0.011), *p* < 0.01, and higher than those for Mandarin (M = 0.715, *SD* = 0.020), and scores for Mandarin were significantly higher than those for English, *p* < 0.01. The main effect of emotion categories was significant [*F* (3, 201) = 43.080, *p* < 0.01, $\eta_{p}^{2}$ = 0.391]. Post-hoc analysis showed that participants scored the highest on the recognition of different emotion categories, happiness (M = 0.770, *SD* = 0.015), and lower than happiness were anger (M = 0.681, *SD* = 0.010), *p* < 0.01, fear (M = 0.670, *SD* = 0.013), *p* < 0.01, and finally sadness (M = 0.635, *SD* = 0.010), *p* < 0.01. The interaction between voice material language and voice emotion categories was significant [*F* (6, 402) = 43.184, *p* < 0.01, $\eta_{p}^{2}$ = 0.045]. Simple effects indicated that Tibetan-speaking adolescents recognized Tibetan more than Mandarin for all the anger, happiness, and sadness emotion categories, with a significant difference for happiness [*F* (2, 66) = 29.855, *p* < 0.01, $\eta_{p}^{2}$ = 0.475]. the difference was not significant on anger (*p* = 0.221), fear (*p* = 1.000) and sadness (*p* = 0.299). The difference was not significant on sadness (*p* = 0.299). Recognition of Mandarin was significantly higher than English on all four emotion categories, anger *F* (2, 66) = 45.403, *p* < 0.01, $\eta_{p}^{2}$ = 0.577; fear *F* (2, 66) = 28.295, *p* < 0.01, $\eta_{p}^{2}$ = 0.462; happiness *F* (2, 66) = 29.855, *p* < 0.01, $\eta_{p}^{2}$ = 0.475; sadness *F* (2, 66) = 42.748, *p* < 0.01, $\eta_{p}^{2}$ = 0.564 (Figure 3B).

##### 4.3.3.2 Response time (RT)

The RT results revealed a significant main effect of language [*F* (2, 134) = 14.767, *p* < 0.01, $\eta_{p}^{2}$ = 0.181]. Post-hoc test showed that emotion recognition for Tibetan material (M = 1258.791, *SD* = 41.926) was significantly faster than English (M = 1586.865, *SD* = 78.401), *p* < 0.01, and faster than Mandarin (M = 1331.490, *SD* = 80.373), as well as significantly faster when responding in Mandarin than in English, *p* < 0.01. There was a significant main effect of emotion categories [*F* (3, 201) = 5.575, *p* < 0.01, $\eta_{p}^{2}$ = 0.077]. Post-hoc analysis showed that participants were fastest when responding to happiness (M = 1254.418, *SD* = 74.273) for the different emotion categories recognized. Fear (M = 1481.293, *SD* = 67.023) was slower than happiness, *p* < 0.01, followed by sadness (M = 1462.454, *SD* = 69.767), *p* < 0.05. Anger (M = 1371.363, *SD* = 65.362) was not significantly different from happiness. There was a significant interaction between voice material language and voice emotion categories [*F* (6, 402) = 4.179, *p* < 0.01, $\eta_{p}^{2}$ = 0.059]. Simple effects analyses showed that Tibetan was faster than Mandarin when responding to anger, fear, happiness, and sadness, with a significant difference on anger and sadness, fear *F* (2, 66) = 9.676, *p* < 0.01, $\eta_{p}^{2}$ = 0.227; sadness *F* (2, 66) = 9.115, *p* < 0.05, $\eta_{p}^{2}$ = 0.216. There were no significant differences in response times for anger (*p* = 0.222) and happiness (*p* = 0.170). Mandarin was faster than English when responding on all four emotion categories, with a significant difference on fear, fear *F* (2, 66) = 9.676, *p* < 0.01, $\eta_{p}^{2}$ = 0.227. There was no significant difference in anger (*p* = 1.000) and happiness (*p* = 0.387) and sadness (*p* = 0.354) (Figure 3B).

##### 4.3.3.3 Mandarin and Tibetan participants comparison in experiment 3

Regarding the results of ACC, a repeated measures ANOVA was conducted on Experiments 3a and 3b with participant group, language and emotion categories as independent variables and accuracy as the dependent variable. As indicated by the results, the main effect of group was significant [*F* (1, 117) = 20.303, *p* < 0.01, $\eta_{p}^{2}$ = 0.148]. According to post-hoc analysis, the emotion recognition scores for Tibetan-speaking adolescents (M = 0.689, *SD* = 0.011) were significantly higher than those for Mandarin adolescents (M = 0.625, *SD* = 0.009), and *p* < 0.01. The interaction between group and voice material language reached a significant extent [*F* (2, 116) = 177.262, *p* < 0.01, and $\eta_{p}^{2}$ = 0.753]. As indicated by simple effects analyses, the Mandarin-speaking adolescents scored (M = 0.807, *SD =* 0.020) significantly higher than the Tibetan-speaking adolescents (M = 0.715, *SD =* 0.017), and *p* < 0.01 for Mandarin. For Tibetan, the Tibetan-speaking adolescents scored significantly higher (M = 0.759, *SD =* 0.011) than the Mandarin-speaking adolescents (M = 0.396, *SD =* 0.013), *p* < 0.01.

As for the results of RT, a repeated measures ANOVA was conducted on Experiment 3a and 3b with participant group, language and emotion categories as independent variables and RT as the dependent variable. According to the results, the main effect of group was significant [*F* (1, 116) = 669.700, *p* < 0.01, and $\eta_{p}^{2}$ = 0.852]. Post-hoc analysis revealed that the emotion recognition scores for Tibetan-speaking adolescents (M = 1380.275, *SD* = 86.192) were significantly faster than those for Mandarin adolescents (M = 2012.579, *SD* = 98.792), and *p* < 0.01. The interaction between group and language was significant [*F* (2, 115) = 52.717, *p* < 0.01, and $\eta_{p}^{2}$ = 0.478]. As indicated by simple effects analyses, for Tibetan, the Tibetan-speaking adolescents scored significantly faster (M = 0.1253.213, *SD =* 128.902) than the Mandarin-speaking adolescents (M = 2897.835, *SD =* 147.745), and *p* < 0.01.

### 4.4 Discussion

Experiment 3a is purposed to investigate how well Mandarin adolescents perform in identifying the vocal emotions in Mandarin, English, and Tibetan. These languages represent different regional cultures within a single nation. The study revealed that Mandarin-speaking adolescents, who did not know Tibetan, exhibited a clear advantage for their own in-group. This is consistent with prior research findings that cultural familiarity influences emotion recognition (Matsumoto and Hwang, 2013). Additionally, the results showed that the adolescents had a greater facility with English, which resulted from educational exposure and media influence.

As revealed by Experiment 3b, compared to Mandarin and English, Tibetan-speaking adolescents exhibited significantly higher recognition rates for vocal emotions in their native language. These findings confirm the presence of an in-group advantage. Growing up within the Tibetan cultural environment, Tibetan-speaking adolescents used Tibetan as their main language for daily communication. Due to this exposure, they become adept at emotional expressions in Tibetan. However, Tibetan-speaking adolescents are relatively weak in recognizing emotional expressions in English, which is attributed to the minimal exposure to English in their cultural and educational surroundings.

## 5 General discussion

In this study, three experiments were conducted to explore how the adolescents from various regions in China perceive voice emotions, involving Mandarin, English, Shaoxing dialect, and Tibetan languages. Experiment 1 confirmed that adolescents exhibited an in-group advantage in cross-cultural voice emotions recognition. In Experiment 2, it was further confirmed that this in-group advantage was possessed by Mandarin-speaking adolescents as well. However, Shaoxing-speaking adolescents showed an in-group advantage in cross-cultural speech emotion recognition compared to English, but this advantage disappeared when compared to Mandarin. That is to say, they failed to perform better in recognizing emotions in Mandarin than in Shaoxing. Finally, it was verified through Experiment 3 that both Mandarin-speaking adolescents and Tibetan-speaking adolescents possessed an in-group advantage in cross-cultural voice emotion recognition. Despite limited research on how adolescents recognize vocal emotions, our study has demonstrated that they have the same ability to recognize cross-cultural vocal emotions as adults (Paulmann and Uskul, 2014; Laukka et al., 2013, 2016; Chronaki et al., 2018).

As indicated by research, it is possible for the individuals learning a second language to encounter challenges in accurately identifying emotions conveyed in non-native vocal cues (Graham et al., 2001; MacIntyre and Vincze, 2017). This is due to the substantial cultural disparities in emotional expressions, phonological attributes, and intonation between English and Mandarin. Despite the exposure to English through education and media, Mandarin-speaking adolescents still lack immersion in an all-English environment. This hinders them from developing their English emotion-recognition skills during language instruction. As a result, they still lack familiarity with English-speaking cultural norms in emotional expression.

As observed in Experiment 2a, Mandarin-speaking adolescents were superior in the recognition of vocal emotions in Mandarin relative to the Shaoxing dialect. However, Experiment 2b showed that Shaoxing-speaking adolescents performed better in recognizing emotions in Mandarin. This results from the widespread use of Mandarin in education. As the official language of China, Mandarin is used in various aspects of life such as education, media, and social interactions. Shaoxing-speaking adolescents, starting to learn Mandarin from a young age, are frequently exposed to the language, which improves their familiarity with emotional expressions in Mandarin (Feng and Adamson, 2012). Additionally, adolescents can develop cognitive patterns for Mandarin as they are at a stage of cognitive development where they are more receptive to new information (Yang and Kong, 2023). In contrast, the Shaoxing dialect is used less frequently in daily life, especially in formal settings, as a result of which their cognitive abilities for that dialect are weaker. Their understanding of emotional expressions in Mandarin is further strengthened by the exposure to Mandarin through literature, films, and television.

It was discovered that the recognition rate of happiness emotions in English among Shaoxing-speaking adolescents was also higher than that in the Shaoxing dialect. It is speculated that there are three possible reasons for this. Firstly, Shaoxing youth begin the exposure to English learning as early as kindergarten. During this time, teachers typically engage students by playing cheerful English nursery rhymes, among other methods. In this way, students can experience the joy conveyed in English, which enhances their ability to recognize happiness emotions in the language (Ambarini, 2012). Secondly, the content of English courses often features the stories and dialogs that express happiness in English. Thus, students are repeatedly exposed to such expressions throughout their learning process, which improves their recognition rate (Ambarini, 2012). Finally, there are often the requirements related to happiness emotions in their course assignments. For instance, adolescents may be asked to watch English comedy films and share their feelings. Because of this direct exposure to the humorous and joyful expressions in English, they are intuitively familiarized with such emotional expressions, which increases their recognition rate for these emotions (Li et al., 2024).

In Experiment 3b, Tibetan-speaking adolescents exhibited a significant in-group advantage for sound emotion recognition. The recognition of all four sound emotions in Tibetan was found higher than that in Mandarin, and their response time was found faster than that in Mandarin. Tibetan-speaking adolescents have been living in Tibet since birth. Immersed in a Tibetan language environment, they communicate in Tibetan with their family members and classmates around them all the time. Also, they have mastered the expression and recognition of various emotions. Even under the policy of popularizing Mandarin, Tibetan was still retained and taught as a subject of study, further reinforcing students’ mastery of the Tibetan language. This resulted in a significant improvement in recognition accuracy for Tibetan compared to Mandarin. Tibetan-speaking adolescents also demonstrated an in-group advantage in recognizing Mandarin and English, with their recognition of Mandarin being superior to that of English. As Mandarin was popularized, they were exposed to and learned it earlier than English. Additionally, Mandarin was used various mainstream media, thus increasing their exposure to the language in learning and entertainment. As for English, which is also one of the participants studied, Tibetan-speaking adolescents may lack sufficient exposure and practice due to limited resources and opportunities. The consequence of this is their lower performance in recognizing emotions in English sounds.

Different dialect groups show variations in the accuracy of recognizing different categories of vocal emotions. For Mandarin-speaking adolescents, the order of recognition accuracy from high to low is as follows: anger > happiness > sadness > fear. For Shaoxing dialect-speaking adolescents, it is: anger > sadness > happiness > fear. For Tibetan-speaking adolescents, it is: happiness > anger > fear > sadness.

There are several limitations to our study. Firstly, only two dialects, Shaoxing and Tibetan, were selected for analysis. Due to historical and geographical influences, the Sino-Tibetan language family has evolved over time, resulting in a wide variety of dialects across different regions. Chinese dialects are typically categorized into 10 major groups, each of which can be further subdivided into the dialects specific to various areas. Additionally, this study focused narrowly on four basic emotions, despite many other social emotions that can be explored in the future. Furthermore, the participants in this study were limited to junior high school students. Therefore, senior high school adolescents should be included in future studies to better understand the emotions in Chinese adolescents’ voices across different regions and cultures. Lastly, the present study was conducted using the recognition accuracy metric rather than the unbiased hit rates (HU scores) as proposed by Wagner (1993). To a certain extent, this methodology has the potential to cause bias in the obtained results. In future research, more precise indicators will be used to explore the voice emotion recognition ability of adolescents, in order to improve the accuracy and reliability of the findings.

## 6 Conclusion

Chinese adolescents were more proficient in identifying vocal emotions from their own cultural group, as opposed to other regional cultures. This advantage was found more pronounced as the cultural distinctions between groups became more significant.

## Data Availability

The datasets presented in this study can be found in online repositories. The names of the repository/repositories and accession number(s) can be found in the article/Supplementary material.
