# Supplementary material for: Cross-regional cultural recognition of adolescent voice emotion
Source: Front Psychol. 2024 Dec 16;15:1437701. doi: 10.3389/fpsyg.2024.1437701 (PMC11683072; doi:10.3389/fpsyg.2024.1437701)
Supplement: Supplementary file 1 [file Table_1.DOCX]

**Table 1. Pseudo sentences in Chinese, English, and Tibetan.**

| **Chinese sentences** | **English sentences** | **Tibetan sentences** |
| --- | --- | --- |
| 她在一个门文上走亮 | Clap the crisp adult lantern. | མོས་སྒོ་ཞིག་གི་སྟེང་དུ་རྩོམ་ཞིག་གི་སྟེང་དུ་བསྐྱོད་། |
| 我们在两上投了一个绳 | Blotch degraded the down dairy snatch. | ང་ཚོས་གཉིས་ཀྱི་སྟེང་དུ་ཐག་པ་གཅིག་འཕངས་། |
| 他们拉摇了我的绳雨 | lambing the jaunted hymns miss. | ཁོ་ཚོས་ངའི་ཐག་པ་འཐེན་ཆར་ |
| 我马上就投量你 | Jagger the daven splint. | ངས་ལམ་སེང་འཕངས་ཚད་ཁྱོད་ཀྱིས་། |
| 他们抄组了一个明平的文柱 | Flop habit the fallen file. | ཁོ་ཚོས་བཤུ་འབྲི་ཚོ་ཆུང་ཞིག་གིས་མིང་ཕིང་གི་ཝུན་ཀྲུའུ་། |
| 我扭了一个非常圆良的春某 | Lyrics the new la liskin. | ངས་ཧ་ཅང་སྒོར་དབྱིབས་ཅན་ཞིག་གཅུས་། |
| 我在腔红之前吐店了 | Dami tan did the dull voile yet. | ངས་ཁོག་དམར་པོ་གོང་སྐྱུག་ཚོང་ཁང་སོང་། |
| 他把雪皮提在广田上 | Flotch deraded the downdary snat. | ཁོས་ཁ་བ་དེ་ཀོང་ཐེན་ཐོག་། |
| 我扶打过这个皮魂 | Ball planned the cacti yelled. | ངས་སྐྱོར་ནས་བརྒྱབ་མྱོང་། ལྤགས་བླ་སྲོག་འདི་ |
| 我将在六馆之后打关 | Gabbard the zestfully. | ངས་ཁང་དྲུག་རྗེས་འགག་སྒོ་བརྒྱབ་། |
| 他在天某里揣写着屯八 | Directs jets, the retina mask. | ཁོང་གིས་ཐེན་ལགས་ཀྱི་ནང་དུ་བརྒྱད་བྲིས་འདུག |
| 她在地泉里提了头山 | Granted the crass blot fascist. | ཁོ་མོས་ས་ཐོག་གི་ཆུ་མིག་ནང་དུ་མགོ་རི་འཐེན། |
| 他把我的铺瓶缠过了 | Contrast juncus, the curl of. | ཁོས་ངའི་ཤེལ་དམ་ལ་འཁྲིལ་སོང་། |
| 他们让我拦了一个夫纸 | Jain did the rendered hinder. | ཁོང་ཚོས་ང་ལ་ཤོག་བུ་ཞིག་བཀག་བྱུང་། |
| 我被他们游讨了 | Clannad the splatter sabotage. | ང་ནི་ཁོ་ཚོས་ཡུལ་སྐོར་འདུལ་སོང་། |
| 他们刨提了我的偶连 | Spins the run sent dupe down. | ཁོང་ཚོས་ངའི་ཟུང་འབྲེལ་གླེང་བྱུང་། |
| 他们在楼谷中投玩 | Flint the runt red papers Gull. | ཁོ་ཚོས་ཁང་བའི་ནང་རྨང་གཞིའི་སྒྲིག་བཀོད་མ་འཇོག་རྩེད་། |
| 我从路瓶里搭乱了 | Hamlet dared the avocado. | ངས་ལམ་གྱི་ཤེལ་དམ་ནང་ནས་རྙོག་སོང་། |
| 她堵摔了一个白丽的平本 | Band red dairy the vogue zoo. | མོས་པའེ་ལི་ཞིག་འགག་ལྷུང་གི་ཕིང་པུན་། |
| 我们在签木中托亮了 | Puzzle wind read the pamper. | ང་ཚོས་མིངརགས་བཀོད་མུའུ་ཀྲུང་ཐུའོ་ལེའང་སོང་། |
| 我的班桃被拢右了 | Janta kettle the goose pads. | ངའི་འཛིན་གའི་ཁམ་བུ་བྱ་ཡུལ་གྱི་གཡས་སོང་། |
| 她昨天洗安了门顿 | Move let trumpet the zilted. | ཁོ་མོས་ཁ་སང་སྒོ་ཏུན་བཀྲུས། |
| 他没有挑跑我们 | Fuller harvest the jumpstart. | ཁོས་ང་ཚོར་མ་འདེམས། |
| 他在我们的窗水里抚摇了 | Phantom rancho red the teasel. | ཁོས་ང་ཚོའི་སྒེའུ་ཁུང་གི་ཆུ་ནང་དུ་ཕུ་འགུལ་སོང་། |
| 他在地上拔冲 | Romanced the topple drill. | ཁོང་གིས་ས་ཐོག་ཏུ་མཆོང་། |
| 我在果体里翻移了桌风 | Soup locked the gula tooth. | ངས་འབྲས་གཟུགས་ནང་དུ་བསྒྱུར་། ཅོག་ཙེ་རླུང་། |
| 他从场东里打了一个头云 | Finter alas the garnish. | ཁོས་ཁྲང་ཏུང་ནས་སྤྲིན་མགོ་ཞིག་བརྒྱབ་སོང་། |
| 筒单里有一个组点 | Import traveler the Carlot. | མདོང་རྐྱང་ནང་དུ་ཚེག་ཚོ་ཆུང་ཞིག་ཡོད་། |
| 他在提哄着一个单雪 | Laurel fissile the skilo. | ཁོང་གིས་ཁ་བ་རྐྱང་ཞིག་ཁྱེར་འདུག |
| 我拐收了一个豆腿 | Can read rent the fiend. | ངའི་རྐང་པ་སྲན་མ་ཞིག་བསྡུས། |
| 我在辉轩里拢饭了 | Bin tread the fine tan valley. | ངས་ཧེ་ཞོན་ནང་ནས་ཟ་མ་ཁྱེར་སོང་། |
| 她在格边里赏了一个天阳 | Putty on the food disposal. | ཁོ་མོས་ཀེ་པན་ནང་དུ་ཉིན་ཞིག་ལ་བྱ་དགའ་སྤྲད། |
| 我把这个空书摇了十个 | Tantalum plaid the banner. | ངས་དེབ་སྟོང་པ་འདི་བཅུ་གཡུགས་། |
| 我在月点里攒了个头文 | Yum the girder habits. | ངས་ཟླ་བའི་ནང་ནས་མགོ་ཞིག་བསགས་། ཝུན་། |
| 他在阔线里踢了米 | Yatade thulium the full promise. | ཁོས་ཞེང་ཐིག་ནང་དུ་རྡོག་པས་བཞུས་། སྨི་། |

The written form of the Shaoxing dialect is identical to Chinese, but the intonation varies.

**Table 2. Participants’ information and their language proficiency**

| **Experiment** | **Permanent Residence** | **Household Registration** | **Gender** | **Native language** | **Mandarin** | **English** | **Shaoxing dialect** | **Tibetan** |
| --- | --- | --- | --- | --- | --- | --- | --- | --- |
| Exp1 | Shandong Province | City | 43 boys, 44 girls | Mandarin | C | B | A | A |
| Exp2a | Shandong Province | City | 19 boys, 25 girls | Mandarin | C | B | A | A |
| Exp2b | Shaoxing City | City | 27boys, 19 girls | Shaoxing dialect | C | B | C | A |
| Exp3a | Shandong Province | City | 28 boys, 23 girls | Mandarin | C | B | A | A |
| Exp3b | Tibet Autonomous Region | City | 37 boys, 31 girls | Tibetan | B | B | A | C |

Level: A: No prior exposure to the language. B: Mastery of some vocabulary, yet incapable of communication. C: Capable of skillful application.
